# A Metabolomics-Based Screening Proposal for Colorectal Cancer

**DOI:** 10.3390/metabo12020110

**Published:** 2022-01-25

**Authors:** Jacopo Troisi, Maria Tafuro, Martina Lombardi, Giovanni Scala, Sean M. Richards, Steven J. K. Symes, Paolo Antonio Ascierto, Paolo Delrio, Fabiana Tatangelo, Carlo Buonerba, Biancamaria Pierri, Pellegrino Cerino

**Affiliations:** 1 Department of Medicine, Surgery and Dentistry “Scuola Medica Salernitana”, University of Salerno, 84081 Baronissi, Italy; 2 Theoreo srl, Via degli Ulivi 3, 84090 Montecorvino Pugliano, Italy; lombardi@theoreosrl.com (M.L.); scala@theoreosrl.com (G.S.); 3 Centro di Referenza Nazionale per l’Analisi e Studio di Correlazione tra Ambiente, Animale e Uomo, Istituto Zooprofilattico Sperimentale del Mezzogiorno, 80055 Portici, Italy; maria.tafuro@izsmportici.it (M.T.); carlo.buonerba@izsmportici.it (C.B.); strategia@izsmportici.it (P.C.); 4 Hosmotic srl, Via R. Bosco 178, 80069 Vico Equense, Italy; 5 Department of Obstetrics and Gynecology, Section on Maternal-Fetal Medicine, University of Tennessee College of Medicine, 960 East Third Street, Suite 100, 902 McCallie Avenue, Chattanooga, TN 37403, USA; seanrichards.utc@gmail.com (S.M.R.); steven-symes@utc.edu (S.J.K.S.); 6 Department of Biology, Geology and Environmental Sciences, University of Tennessee at Chattanooga, 615 McCallie Ave., Chattanooga, TN 37403, USA; 7 Department of Chemistry and Physics, University of Tennessee at Chattanooga, 615 McCallie Ave., Chattanooga, TN 37403, USA; 8 Istituto Nazionale Tumori Fondazione Pascale IRCCS, 80131 Napoli, Italy; p.ascierto@istitutotumori.na.it (P.A.A.); p.delrio@istitutotumori.na.it (P.D.); f.tatangelo@istitutotumori.na.it (F.T.)

**Keywords:** metabolomics, colorectal cancer, screening test, fecal occult blood test, ensemble machine learning

## Abstract

Colorectal cancer (CRC) is a high incidence disease, characterized by high morbidity and mortality rates. Early diagnosis remains challenging because fecal occult blood screening tests have performed sub-optimally, especially due to hemorrhoidal, inflammatory, and vascular diseases, while colonoscopy is invasive and requires a medical setting to be performed. The objective of the present study was to determine if serum metabolomic profiles could be used to develop a novel screening approach for colorectal cancer. Furthermore, the study evaluated the metabolic alterations associated with the disease. Untargeted serum metabolomic profiles were collected from 100 CRC subjects, 50 healthy controls, and 50 individuals with benign colorectal disease. Different machine learning models, as well as an ensemble model based on a voting scheme, were built to discern CRC patients from CTRLs. The ensemble model correctly classified all CRC and CTRL subjects (accuracy = 100%) using a random subset of the cohort as a test set. Relevant metabolites were examined in a metabolite-set enrichment analysis, revealing differences in patients and controls primarily associated with cell glucose metabolism. These results support a potential use of the metabolomic signature as a non-invasive screening tool for CRC. Moreover, metabolic pathway analysis can provide valuable information to enhance understanding of the pathophysiological mechanisms underlying cancer. Further studies with larger cohorts, including blind trials, could potentially validate the reported results.

## 1. Introduction

Colorectal cancer (CRC) represents approximately 10% of all annually diagnosed cancers and cancer-related deaths worldwide. It is the second most frequent cancer diagnosed in women and the third most in men [1]. Its incidence is higher in developed countries. However, while screening programs and improved lifestyle habits help stabilize incidence in these countries, the global incidence is expected to increase as affluence increases and lifestyle changes in developing countries [2].

Both hereditary and environmental risk factors play a role in the onset of CRC. Family history of CRC is a known risk factor for developing the disease and encompasses both genetic and shared environmental risk factors [3]. Among familial cases, a subgroup of patients (3–5% of all CRC [4]) is affected by hereditary CRC syndrome, which can be subdivided into non-polyposis (such as Lynch syndrome) and polyposis syndromes, such as Peutz–Jeghers syndrome and familial adenomatous polyposis (FAP) [5]. Known modifiable environmental risk factors include smoking, eating processed and red meat, alcohol intake, low intake of vegetables and fruits, and obesity [6]. Among non-modifiable factors, male sex, age, and inflammatory bowel diseases such as ulcerative colitis are associated with an increased risk of developing CRC [7]. 

Clinical signs and symptoms associated with CRC are occult or overt rectal bleeding, change in bowel habits, anemia, weight loss, and abdominal pain. However, they are not specific for this condition. Unfortunately, CRC may be asymptomatic until it reaches advanced stages [8]. 

Secondary prevention, implemented through screening programs aimed at an early diagnosis, is of crucial importance for CRC, for several reasons. First, the most important prognostic factor is disease stage at diagnosis. Indeed, in the USA in the period 2001–07, 5-year survival was 90.1% for patients with localized stage, 69.2% for patients with regional spread, and 11.7% for patients with distant tumor spread [9]. Second, CRC mostly progresses from precursor lesions (mainly polypoid) by the sequential accumulation of genetic mutations and epigenetic alterations, over a mean progression period of 10–15 years [10]. Thus, the ideal method of screening should be able to detect pre-neoplastic lesions or, at least, localized neoplastic lesions, to allow radical and resolutive intervention. 

At present, CRC surveillance is based on invasive, i.e., colonoscopy, and non-invasive methods, i.e., fecal occult blood test, targeting either heme (guaiac fecal occult blood test-gFOBT) or hemoglobin (fecal immunochemical test; hemoglobin-FIT). A meta-analysis of four randomized controlled trials concluded that annual or biennial gFOBT screening had no effect on CRC incidence but led to an average 16% mortality reduction [11]. No randomized controlled trial has reported the impact of FIT screening on CRC incidence and mortality; however, the latter method is preferred over the former because it is more sensitive. Nevertheless, as with all screening tests, FIT diagnostic performance depends on the cutoff value for a positive test result [12]. 

Colonoscopy is, at present, the best method to screen for CRC, in terms of specificity and sensitivity [13]. Nevertheless, randomized controlled trials aimed at quantifying the impact of colonoscopy screening on disease outcomes are still ongoing (clinical trial numbers: NCT01239082, NCT00883792, NCT02078804). An advantage is that colonoscopy offers the possibility of direct lesion removal [13]; however, it is invasive and requires adequate bowel preparation in addition to highly trained personnel.

Alternative screening methods are sigmoidoscopy and computed tomography (CT) colonography. Four large randomized controlled trials on sigmoidoscopy screening have been done. All studies showed a reduced incidence of colorectal cancer and, three of four resulted in a lowered relative mortality risk as well [14,15,16,17]. CT colonography has comparable accuracy with respect to colonoscopy [18]. However, both of these are often two-step screening methods, because they must also perform a total colonoscopy in any case in which further diagnosis is needed. 

In spite of the wide range of screening options currently available for CRC, such tests remain under-utilized by the public [19]. In addition to low public awareness of the importance of screening programs, psychological factors play a key role in determining how often CRC screening is performed. Invasive procedures, such as colonoscopy, are often rejected due to the fear of pain, complications, and discomfort. On the contrary, stool-based approaches are generally preferred due to lower costs and invasiveness, but the need for multiple tests represents a major discouraging factor for many [20]. Even though utilization of invasive procedures is significantly lower than that of stool-based methods, overall compliance with the latter remains low because people tend to postpone these investigations, despite the potentially crucial loss of time before being screened [21]. 

However, as the adherence of the target population to screening programs is pivotal to obtaining successful public health results, the need to address this issue becomes prominent. Hence, significant effort is expended to develop novel diagnostic tools in order to encourage and simplify the screening of CRC, including, as an example, the development of a toilet paper-based FOBT method [22]. In this context, metabolomics analysis may offer valuable support. 

Metabolomics is an emerging field of research in the -omic domain and refers to a comprehensive analysis of low molecular weight compounds, such as metabolic substrates and products, lipids, small peptides, vitamins, and other protein cofactors, generated by metabolism, in a biological fluid. It is a rapidly growing field in biomarker discovery [23]. Moreover, unlike the other -omic sciences, such as genomics, transcriptomics, and proteomics, it can be more precise in the characterization of multifactorial diseases because it reflects the interactions between genes and the environment [23,24].

Compared to other diagnostic tools, the metabolomics approach can offer high diagnostic performance by means of a single analysis, in a cheap, fast, and non-invasive manner, potentially representing the ideal screening test. Furthermore, as the metabolome provides unique information regarding the mechanisms underlying the disease onset and progression, a thorough investigation of the metabolomic fingerprint of CRC may provide crucial insights to enhance the understanding of the pathology as well as to identify prognostic biomarkers and assess the severity of the disease [25]. 

Several metabolomic studies have been conducted on a variety of biological matrices (blood, urine, fecal water, tissue) in small cohorts of colorectal cancer patients [26,27]. These compared either metabolic profiles to healthy subjects (and to normal tissue samples) [26] or to patients with benign polypoid pathology [28], using gas and liquid chromatography coupled to mass spectrometry (GC-MS, LC-MS) or nuclear magnetic resonance (NMR) as analytical techniques. 

Here, we describe the results of an untargeted metabolomics-based profiling of serum samples collected from subjects that tested positive using the FOBT screening program. Stratifying them according to their colonoscopy-based biopsies, the population was divided into three groups: healthy subjects, participants with benign colon lesions, and patients with CRC. The specific aim was to propose a novel, non-invasive method for the screening of CRC using a robust ensemble machine learning approach based on serum metabolomes.

## 2. Results

The reported results were achieved by analyzing serum samples taken from 200 individuals who tested negative for FOBT or positive and subsequently underwent a colonoscopy and a biopsy to pathologically analyze any evident lesions. Fifty of the 200 participants presented with no lesions and were considered healthy subjects (HS), 50 presented with benign colon or rectum tumors (BCRT), and 100 were diagnosed with CRC.

Gas chromatography–mass spectrometry analysis of derivatized samples detected up to 261 peaks in each specimen using an untargeted metabolite extraction procedure. Peaks present in at least 75% of samples and with sufficient signal to be confirmed as metabolites using library comparison were further investigated. As a result, a total of 243 signals were consistently detected. Supplementary Figure S1 reports the deconvoluted chromatograms of typical CTRL and CRC samples.

For all of the enrolled subjects, age, sex, weight, height, and biochemical parameters results were recorded. Moreover, the presence of other pathological conditions, as well as chronic treatments for these conditions were investigated (Table 1). These parameters were normally distributed according to the Shapiro–Wilk test. All statistical comparisons used a significance value of α = 0.05 (described in detail below). CRC patients were significantly older than HS subjects (*p* = 0.009), whereas CRC patients showed a lesser mean body mass index (BMI) compared to both HS and BCRT subjects (*p* < 0.001). 

For the purpose of attempting to distinguish serum metabolomes of cancer vs. non-cancer subjects, the HS and BCRT groups were combined to form the control (CTRL) group. Based on this sample aggregation, training and test sets were prepared by randomly dividing the overall dataset (N = 200) into two parts (66:34). One (*n* = 133; composed of 69 CTRL and 64 CRC) was used to train and cross-validate multiple classification models, while the other (*n* = 67; with 31 CTRL and 36 CRC) was used to test them. Overall classification performance was evaluated using the test set.

In total, 86,625 models, based on 25,141 feature subsets, were developed and tested with the aim of determining the most effective combination of hyperparameters and metabolites to maximize the accuracy of classification of the examined models. Ten machine learning algorithms were trained to classify samples as CTRL or CRC based on the metabolomic profile. These include naïve Bayes (NB), generalized linear model (GLM), logistic regression (LR), fast large margin (FLM), deep learning (DL), decision tree (DT), random forest (RF), gradient boosted tree (GBT), support vector machine (SVM), and partial least square discriminant analysis (PLS-DA). Ultimately, individual results from the ten classification models were statistically “ensembled” to generate an ensemble machine learning algorithm (EML). The best metabolite subsets used for training the final models, as selected by a genetic algorithm (GA), are reported in Supplementary Table S1. The table also reports the metabolites with the highest weight used to build the UpSet representation.

**Figure 1:**
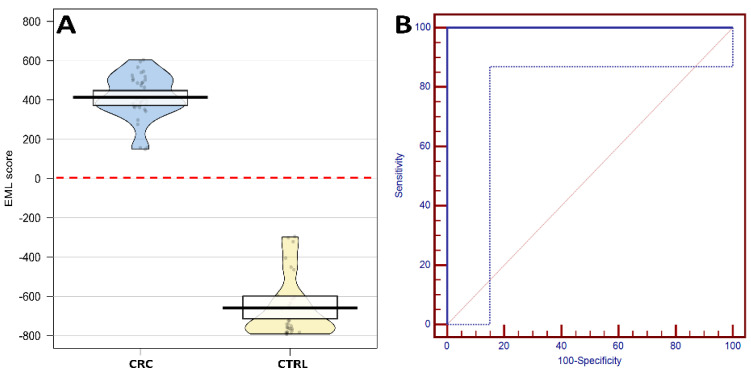
Ensemble machine learning (EML) scores calculated for the healthy controls (CTRL) and patients with colorectal cancer (CRC) among the test set; red dashed line represents the optimized cut-off value (Panel **A**). Receiver operating characteristic (ROC) curve obtained by varying the cut-off value when applying the EML model to the test set (Panel **B**); the area under the ROC curve is 1.0. Dotted blue line represents the 95% Confidence Bounds.

As highlighted in Table 2, individual model classification accuracy ranged from 71% to 100% while the EML model reported no classification errors, resulting in 100% accuracy. For EML score evaluation, an EML score = 0 was selected as the optimized cut-off value and represents situations in which the individual votes for and against CRC-positive diagnosis were equal. Figure 1 reports the EML score distribution among the samples in the test set and the corresponding ROC curve. Supplementary Table S2 reports the classification results as well as the classification confidence for the enrolled samples among the test set.

**Figure 2:**
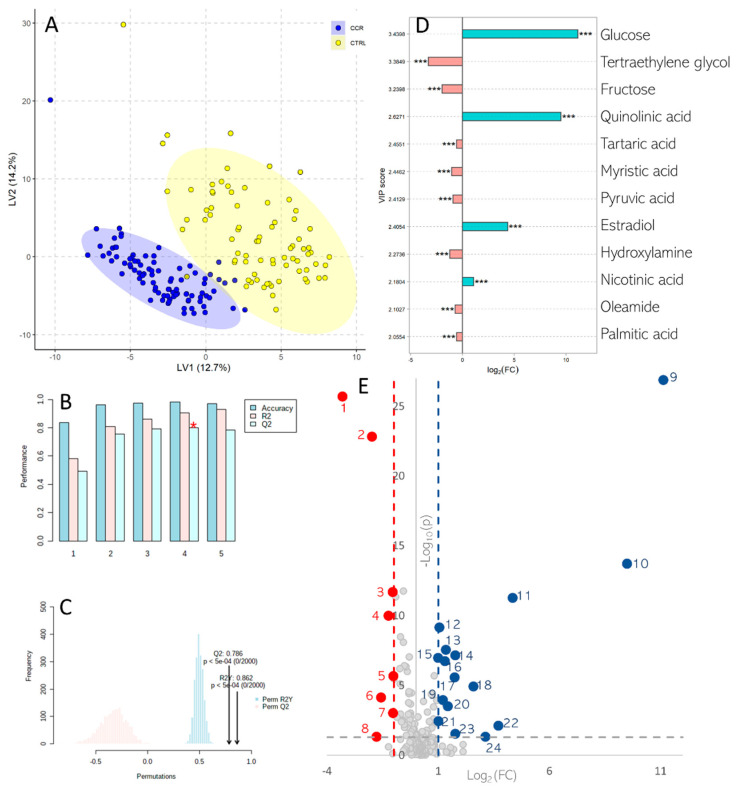
Partial least square discriminant analysis (PLS-DA) score plot performed to classify CTRL and CRC subjects (panel **A**). For each axis, the percentage of explained variance is reported in parentheses. Panel (**B**) reports the PLS-DA classification performance using increasing number of latent variables. The red star indicates the best classifier. (**C**) Permutation test results in which models were built by randomly assigning the class label and then comparing the performance of the permuted models with that of the original model built with the correct class assignment. These were statistically different (based on 2000 permutations), highlighting the lack of overfitting in the original model. (**D**) The metabolites showing a variable importance in projection (VIP) score higher than 2.0. The blue bars represent metabolites increased in CTRL, while the red bars represent the metabolites decreased in CTRL with respect to CRC. *** represent metabolites with a *p*-value < 0.001 (**E**) Volcano plot reporting metabolite concentration fold-changes and their statistical significance when comparing CTRL vs. CRC subjects. 1. Galactose, 2. 4-Hydroxybenzyl alcohol, 3. Myristic acid, 4. Hydroxylamine, 5. Arabinose, 6. Guanine, 7. Fructose, 8. Tetraethylene glycol, 9. Glucose, 10. Quinolinic acid, 11. Estradiol, 12. Threonine, 13. Glutamine, 14. Glyceryl-glycoside, 15. Oxoproline, 16. Lactose, 17. Oxoglutaric acid, 18. 2-Ketobutyric acid, 19. Mandelic acid, 20. Creatinine, 21. Glutamic acid, 22. Nicotinic acid, 23. Norepinephrine, 24. Acetic acid. Horizontal dashed grey line shows *p* = 0.05; vertical dashed lines represent log_2_FC = ±1.

The PLS-DA scatter plots of the first two latent components, reported in Figure 2A, show the graphical representation of the class separation achieved between CTRL and CRC samples. The model showed the best performance using four latent components (Figure 2B) and was statistically robust with no overfitting as confirmed by the permutation test represented by a histogram plot presented in Figure 2C. Fitting value R^2^ and its cross-validation homolog Q^2^ were 0.907 and 0.787, respectively. Twelve metabolites were found to be most relevant to the class separation (as determined by a variable importance in projection (VIP) score >2.0). These metabolites, reported in Figure 2D, were: glucose, tertraethylene glycol, fructose, quinolinic acid, tartaric acid, myristic acid, pyruvic acid, estradiol, hydroxylamine, nicotinic acid, oleamide, and palmitic acid. 

The exploratory analysis illustrated in the volcano plot of Figure 2E showed that 24 metabolites displayed both large magnitude fold-changes (2 < FC < 0.5) and high statistical significance (*p* < 0.05) when comparing CTRL vs. CRC among the 200 enrolled subjects. Of these, glucose, quinolinic acid, estradiol, threonine, glutamine, glyceryl-glycoside, oxyproline, lactose, oxoglutaric acid, 2-ketobutyric acid, mandelic acid, creatinine, glutamic acid, nicotinic acid, norepinephrine, and acetic acid were higher in CTRL compared to CRC. Conversely, galactose, 4-hydroxybenzyl alcohol, myristic acid, hydroxylamine, arabinose, guanine, fructose, and tetraethylene glycol were higher in CRC.

**Figure 3:**
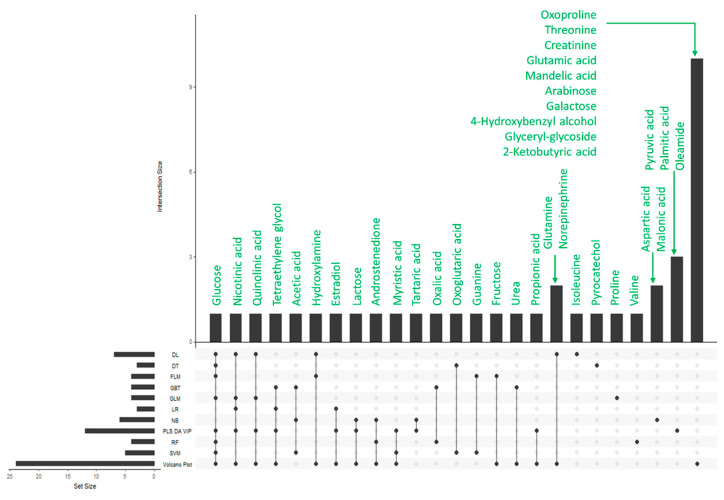
UpSet representation showing the metabolites selected as significant by a given classification model (horizontal) in addition to multiple models selecting a given metabolite (vertical).

The statistical significance of each metabolite in model training was evaluated for all of the classification algorithms. Those that were found to be most relevant within a given model combined with their multiple selections (in several classification models) were summarized using an UpSet diagram reported in Figure 3. All selected metabolites were coded according to the Human Metabolites Database (HMDB) and reported in Supplementary Table S2. Supplementary Figure S2 reports the box and whisker plot representation of the relative abundances of the relevant metabolites according to the raw signals and the transformed data.

**Figure 4:**
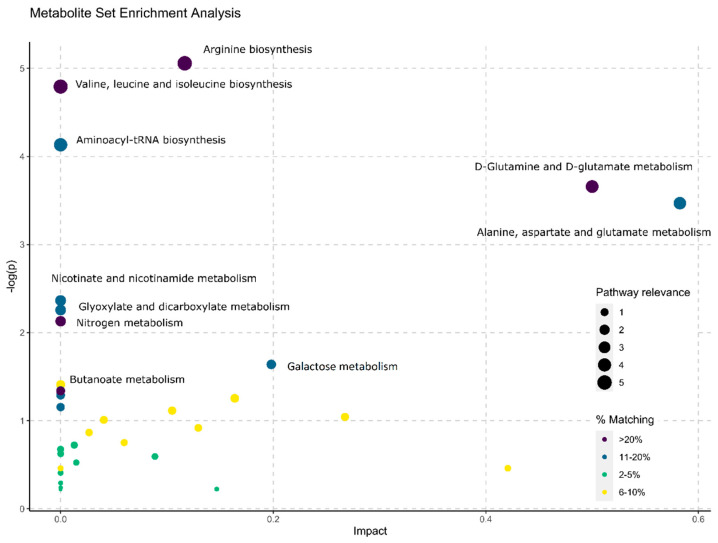
Metabolite set enrichment analysis establishes whether compounds implicated in a specific pathway are increased compared to casual occurrence applying the hypergeometric test. Node centrality, which represents an estimate of node importance, was achieved by 0 employing the betweenness centrality. This reveals the number of shortest paths passing through the node. Because the metabolic network is directed, the relative betweenness centrality for a metabolite has been applied as the importance measure. The betweenness centrality measure is focused on the total network topology. Pathway relevance (represented in terms of circle size) was evaluated as the distance of each point (a metabolic pathway) from the axis origin. Colors represent the matching status of each pathway (number of reported metabolites compared to the total metabolites in the pathway).

These metabolites were also employed to conduct a metabolite-set enrichment analysis, reported in Figure 4. An intricate interplay of a number of different metabolic pathways and metabolites was found. For example: arginine biosynthesis; valine, leucine, and isoleucine biosynthesis; aminoacyl-tRNA biosynthesis; D-glutamine and D-glutamate metabolism; alanine, aspartate, and glutamate metabolism; nicotinate and nicotinamide metabolism; glyoxylate and dicarboxylate metabolism; nitrogen metabolism; galactose metabolism; galactose metabolism and butanoate metabolism were all found to play a role in distinguishing the serum metabolomes of CTRL from CRC subjects. The complete list of involved pathways can be found in Supplementary Table S3.

## 3. Discussion

In the present study, we evaluated the diagnostic performance of a machine learning ensemble model based on the statistical comparison of the serum metabolomic fingerprints of individuals that are and are not affected by malignant colorectal cancer. Univariate and multivariate comparison of hundreds of serum metabolites illustrates biochemical discrepancies in serum samples according to the presence or absence of CRC. 

According to the results, numerous serum metabolites and metabolic pathways seem to be associated with CRC. The analysis of the complex network of metabolic pathways connecting molecules, in combination with a powerful machine learning algorithm, enables an effective group separation, offering an innovative approach for noninvasive CRC screening, and providing useful biochemical insights regarding the involved metabolomic pathways. In particular, lower levels of glucose and glutamine were observed in CRC patients. In cancer cells, glucose and glutamine represent the most consumed nutrients [29]. Moreover, both glutaminase and glutamate dehydrogenase are overexpressed in many cancers [30,31]. Notably, glutamine modulates glutaminolysis. This, in combination with leucine, which is capable of activating glutamate dehydrogenase, induces α-ketoglutarate production preventing glutaminase inhibition by glutamate accumulation [32].

Line et al. [33] reported that low glutamine amounts were associated with older age, advanced-stage cancer, low albumin levels, high carcinoembryonic antigen levels, increased C-reactive protein levels, higher modified Glasgow prognostic scores, and augmented proinflammatory cytokine levels in colorectal cancer-affected patients. Moreover, according to their results, subjects exhibiting decreased glutamine levels had lower overall survival and progression-free survival compared to individuals with higher glutamine levels. 

The low levels of glutamine are consistent with the well-known “glutamine addiction” of cancer cells [34] reported in bladder cancer [35], lung cancer [36], and glioma [37]. This is due to the need for cancer cells to sustain the production of acetyl-coenzyme A to synthetize fatty acids when pyruvate is converted to lactate. 

The low levels of aspartic acid reported here could explain the low levels of quinolinic and nicotinic acid levels because these molecules are produced from aspartic acid. The involvement of aspartic acid in CRC was reported several times although with different concentration changes in both serum and feces, highlighting its different role in advanced and early stages of the disease [38]. The pivotal role of aspartic acid was also highlighted by Nishiumi et al. [39] who proposed a four metabolite-based (2-hydroxybutyrate, aspartic acid, kynurenine, and cystamine) screening test to discriminate CRC patients from controls with AUC-ROC of 0.91 and an accuracy of 85%. Nicotinate and nicotinamide metabolism as well as leucine, isoleucine, and valine metabolism; short-chain fatty acid and alanine and aspartate metabolism were shown to have a significant impact on CRC metabolomics signature. Brown et al. [40] also found that nicotinate and nicotinamide metabolism was associated with a CRC metabolomics signature.

The higher abundances of tetraethylene glycol and hydroxylamine observed in CRC subjects may be caused by an increased exposure to these molecules or the metabolism of correlated pollutants. The selection of non-smoking subjects, both among CRC patients and healthy controls, allows us to exclude these substances as being related to smoking, although passive smoking exposure was not deeply investigated.

In addition, the involvement of short-chain fatty acids (SCFA) including acetic, propionic, and 2-keto butyric acids might be explained by differences in microbiome composition between patients suffering from CRC and healthy controls. Indeed, evidence exists that microbiomes play a crucial role in CRC. In particular, several studies [41,42,43,44] modulating several host mechanisms, ranging from inflammation to DNA damage, and producing metabolites able to modulate cellular transformation and cancer progression or suppression. Gut microbiota imbalance (aka dysbiosis) has been reported in subjects affected by CRC, who exhibit lower counts of commensal bacteria (especially butyrate-producing species) and increased levels of pro-inflammatory opportunistic pathogens. The low concentrations of SCFA reported herein could be interpreted in line with this evidence. 

The reduced concentration of 4-hydroxybenzyl alcohol reported herein has an established correlation in CRC metabolomics-based studies [40,45]. This metabolite shows good angiogenetic antagonist activity [46] and was decreased in polyvinyl chloride workers with hepatic hemangiosarcoma [47]. 4-hydroxybenzyl alcohol, (aka gastrodigenin), is an intermediate metabolite produced during the biosynthesis of thiamine by *Escherichia coli*. Luo et al. [48] reported that the anti-angiogenic and anti-tumor effects of 4-hydroxybenzyl alcohol could, in part, be due to upregulation of nuclear factor erythroid 2-related factor 2 (Nrf2), an emerging regulator of cellular resistance to oxidants, and in part to other antioxidant pathways. Moreover, this result could also be related to the already discussed CRC-derived dysbiosis. 

Lipid involvement in cancer and in particular, colorectal cancer, is established [49]. Li et al. [50] reported a nine biomarker-based panel, containing palmitic amide, oleamide, hexadecanedioic acid, octadecanoic acid, eicosatrienoic acid, LPC (18:2), LPC (20:4), LPC (22:6), myristic acid, and LPC (16:0), for an effective differentiation of early-stage patients from healthy controls (AUC-ROC = 0.991, sensitivity = 0.981 and specificity = 1.000). Herein, we observed high levels of oleamide in serum samples of CRC patients. This endogenous metabolite is known to accumulate in the cerebrospinal fluid during sleep deprivation and induces sleep in animals [51]. The exact mechanism of action of oleamide’s sleep-inducing effects is still to be completely elucidated; however, it is likely that oleamide interacts with multiple neurotransmitter systems. Oleamide is structurally related to the endogenous cannabinoid anandamide and has the ability to bind to the CB1 receptor as a full agonist. The higher concentrations reported herein could reflect cannabinoid receptor expression increase in CRC patients [52].

Overall, the CRC signature seems in part related to the Warburg effect (i.e., altered energetic metabolism of cancer cells to facilitate growth, survival, and proliferation), and specifically, the glutamine addiction reported in several cancer types. These well-established effects may help explain the high sensitivity of the ensemble machine learning model built herein using the serum signature. The other part of the CRC signature seems to be related to more specific effects such as the SCFA imbalance, the lipid profile, especially involving oleamide, and gastrodigenin. These differences may contribute to the high degree of CRC specificity of the serum signature and could lead to a differentiation with other oncological forms. This aspect was not part of our experimental design and represents the weakness of our study.

The diagnostic performances of the classification models described herein, taken independently, are comparable to other studies reported in the literature. As an example, the sensitivity and specificity of PLS-DA we reported (92% and 87%, respectively) are comparable to those reported by Farshidfar et al. [53] (85% and 86%, respectively), as well as the results of the minimal panel reported by Li et al. [50] (98% and 100%). The strength of our approach lies in the EML algorithm. Indeed, all individual models (except DL) made some classification errors on the test set. On the contrary, the ensemble did not make any errors because more than half of the models would have to make the same mistake on the same sample for EML to show a classification error. This makes the EML system more robust compared to using a single classification approach and contributes to the observed high performance of the ensemble approach.

Nevertheless, our study must only be considered a pilot study. Studies based on a single population can be affected by population selection biases as well as analytical biases. Despite efforts to exclude such biases, and the use of cross-validation and permutation tests to avoid overfittings, this risk cannot be completely excluded. Independent validation from a larger, multi-centric, blind cohort is imperative to probe the diagnostic performance under real-world conditions. In this way, it could be investigated whether confounding factors (other age range subjects, concurrent pathologies, etc.) contribute to limiting its applicability or diminish its overall performance. 

## 4. Materials and Methods

### 4.1. Study Design and Patients Enrollment

The clinical specimens have been collected within the “Prima Prevenzione—SPEM” study protocol—“Analysis of environmental, dietary, transcriptomic and genomic factors as biomarkers for risk assessment and early diagnosis of colorectal cancer within the screening campaign “Prima Prevenzione”, approved by the Ethics Committee “Campania Sud” (approval n° n.144, 6 December 2018). The “Prima Prevenzione—SPEM” study is an epidemiological observational cohort study promoted by the Istituto Zooprofilattico Sperimentale del Mezzogiorno (IZSM) of Portici (Naples), in collaboration with the Local Health Authorities of Salerno (ASL SA) and the National Tumor Institute IRCCS (Istituto di Ricovero e Cura a Carattere Scientifico) “G. Pascale” in Naples. The study design considers an integrated and holistic search for a predictive profile of the occurrence risk of CRC. It takes into account the individual hereditary/etiological causes and their combination, especially focusing on the correlation between environment and health and an innovative perspective of primary (prophylactic interventions) and secondary (early diagnosis) prevention. There are three main study areas: (1) Genomic, epigenomic, and metabolomics biomarkers; (2) exposure to sources of pollution; (3) eating habits and intestinal microbiota.

About 60,000 subjects were screened for FOBT; the positive ones were subjected to colonoscopy and eventually to colon mucosa biopsy to confirm the presence of colorectal cancer. Inclusion criteria were: (1) age between 50 and 74 years; (2) residence in the municipality of enrollment for at least 5 years (in order to standardize environmental exposure); (3) history of negative hepatitis B, C and HIV; (4) signature of the informed. Exclusion criteria were: (1) Contraindication to blood sampling; (2) lack of cooperation or poor compliance. Among the enrolled subjects, 200 non-smokers were randomly selected resulting in 3 study groups:

(I) Control Healthy Group: Negative or positive fecal occult blood test (FOBT−/+) with a negative endoscopy (*n* = 50);

(II) Benign Colorectal Disease Group: Positive fecal occult blood test (FOBT +) with a positive endoscopy for benign adenomatous polyp without carcinoma (*n* = 50);

(III) CRC Group: Positive fecal occult blood test (FOBT +) with a positive endoscopy and positive histology for CRC (pT1-2 N0 or pT3-4N0 or PT1-4 N +) (*n* = 100).

After subscribing to an informed consent, each subject enrolled in the study first underwent medical examination, completed a questionnaire on lifestyle and eating habits (EPIC questionnaire—European Prospective Investigation into Cancer and Nutrition—validated by WHO—World Health Organization) [54,55], and filled in an anamnestic case report form (CRF). Then, samples of blood (about 50 mL), urine, and feces were collected. All data were treated in accordance with current legislation on privacy (EU 679/2016). Indeed, data encryption was applied in order to ensure the protection of privacy on the web-based platform for the study management (https://pps.openspes.campaniatrasparente.it/, accessed on 2 December 2021).

### 4.2. Blood Sampling

Blood samples were collected in vacutainer tubes for serum (BD REF 366,468 SST II Advance Tube) and separated by centrifugation at +4 °C, 10 min at 2000 RCF. Fresh samples were allocated to biochemical analysis, within 3 h from venous sampling. The remaining aliquots were stored at −80 °C in the biobank dedicated to the study (www.biobancaizsm.it, accessed on 1 December 2021), until analysis of biomarkers.

The clinical and biochemical parameters defining a basal profile of the individuals were evaluated. These included blood count, blood biochemistry, and endocrinological panel. These were considered in order to investigate the function of bone marrow, liver, kidney, and of the endocrine system and to underline the presence of clinically relevant differences among the three study groups.

### 4.3. Metabolomics Analysis

Untargeted extraction, purification, and derivatization of serum samples were performed using MetaboPrep GC kit (Theoreo srl, Montecorvino Pugliano, Italy) as described in Troisi et al. [56,57]. Briefly, 50 µL of serum was placed in an Eppendorf tube and incubated with the alcohol-based extraction solution containing 2-isopropyl malic acid as internal standard. Tubes were vortexed at 1250 rpm for 30 min and subsequently centrifuged for 5 min at 16,000 rpm at 4 °C; 200 µL of supernatants, collected in new tubes were incubated under vortex conditions (1250 rpm for 30 s) with the purification solution and then centrifuged at 16,000 rpm at 4 °C for 5 min. Supernatants (175 µL) were transferred into a glass vial and freeze-dried overnight. Derivatization was conducted in two steps: First, a pyridine solution of methoxylamine was added and solutions were incubated under vortexed conditions (1200 rpm for 90 min); subsequently, 25 µL of an N,O-Bis(trimethylsilyl)trifluoroacetamide (BSTFA)-based derivatizing solution was added. Vials were subjected to an additional 90 min vortexing at 1200 rpm.

The derivatized metabolome was transferred to a GC vial with a low-volume insert for the autosampler injection. Vials were centrifuged for 5 min at 16,000 rpm maintaining the temperature below 4 °C, before the injection into GC-MS.

Derivatized samples (2 µL) were injected into the GCMS-2010SE (Shimadzu Corp., Kyoto, Japan). The chromatographic separation was performed using a 30 m × 0.25 mm CP-Sil 8 CB fused silica capillary column with 1.00 µm film thickness from Agilent (Agilent, J&W). Helium was used as carrier gas; the initial oven temperature was set at 100 °C and was maintained for 1 min and subsequently raised to 320 °C at 6 °C/min with a further hold time of 2.33 min. The gas flow was set to reach a constant linear speed of 39 cm/s, and the split flow was set to 1:5. The mass spectrometer was operated with electron impact ionization (70 eV) in full scan mode with a range of 35–600 m/z, a scanning speed of 3333 amu/sec, and a solvent cut-time of 5 min. Relevant metabolites were annotated setting to 50 the linear index difference max tolerance and setting to 85% the minimum matching for NIST-14 library search. These were further confirmed using external standards according to Level 1 Metabolomics Standard Initiatives (MSI) annotation [58].

The samples were partitioned into batches, each consisting of 25 samples. Four controls were used to monitor each batch: an instrument blank injection, an injection of a standard mixture, an injection of a pooled sample solution, and a duplicated injection of a randomly chosen sample in the batch. In particular, 2 µL of hexane was employed for the instrument blank, while the standard mixture contained a solution of 15 molecules (organic acids, sugars, amino acids, steroids, and fatty acids) that underwent the same derivatization process used for the samples. The pooled sample consisted of 2 µL each from 50 randomly selected derivatized samples, and the duplicated injection was performed employing a sample chosen at random from the batch.

Four conditions had to be met for each batch to be validated: No peaks were generated by the solvent blank; the ratio between the areas underlying the peak of the analytical standards (normalized by the internal standard area) remained within 10% of the expected value; the peak areas (normalized to the internal standard) of the 100 highest peaks of the repeated injection were within 15% of the first injection; and the pooled sample was allocated in the same area of the other pooled samples; that is, <5% of the total area of a model built using all the samples analyzed.

For each batch, an alkane mixture (C10-C40, Sigma-Aldrich, Milan, Italy) was injected to evaluate the Kovats’ index [59]. Moreover, for each batch, an injector liner change was performed. Injection liners contained a small amount of GC-MS-grade glass wool.

Gas chromatography–mass spectrometry signals not consistently found in at least 80% of the samples were excluded. Very low-intensity metabolite peaks, resulting from low concentration and therefore poor mass spectral quality, were not investigated further. Signals derived from the same metabolites (e.g., sugars that result in multiple derivatization products) were considered as independent features.

Chromatographic signals were first deconvoluted and peaks were picked using the GC-MS Solution software v.2.72 (Shimadzu, Kyoto, Japan) and then aligned using the MetaboPredict software (Theoreo srl, Montecorvino Pugliano, Italy), which uses the *ptw* R package [60] within a proprietary script for gas chromatographic data alignment and missing data management.

### 4.4. Statistical Analysis

Statistical analyses were performed by means of R Studio ver. 1.2.5042. The Shapiro–Wilk test was used to analyze clinical data distribution. Because the continuous variables were normally distributed, *p*-values were determined using the Student’s *t*-test, whereas the comparison of percentages was achieved using the χ^2^-test. Statistical significance was evaluated using α = 0.05. With regard to bioinformatic analysis, data obtained by the chromatographic investigations were compiled in a table with one sample per row and one variable (metabolite) per column (dataset). Data transformation of the raw chromatographic signal intensities was performed by first taking the logarithm of the peak areas for each metabolite (normalized to that of the internal standard) and then scaling these values via the autoscaling process (mean centered and divided by the standard deviation for that variable).

### 4.5. Machine Learning Models

After samples were separated into training (*n* = 133) and test (*n* = 67) sets (66:34 ratio), the first set was employed to train 10 classification models: Naïve Bayes (NB), generalized linear model (GLM), logistic regression (LR), fast large margin (FLM), deep learning (DL), decision tree (DT), random forest (RF), gradient boosted trees (GBT), support vector machine (SVM), and partial least square discriminant analysis (PLS-DA). Fine hyperparameter tuning was implemented to reach the optimal combination in order to maximize the classification accuracy of the models and avoid overfitting (evaluated by means of a cross-validation procedure on the training dataset). Meanwhile, numerous metabolite subsets were used to train the models.

As suggested by the software, we used for data analysis, Rapid Miner Studio ver. 9.7.0 (RapidMiner GmbH, Boston, MA, USA), features to be included in the model were screened in accordance with three criteria: (a) correlation (features that too closely, or not at all, mirror the Yes/No diagnosis criterion), (b) stability (features where nearly all values are identical), (c) missing (features with missing values) and then mixed to identify the combination providing the best performance. Moreover, a metabolites subset selection was applied to each classification model as a nested genetic algorithm (GA) using the evolutionary features selection tool included in the RapidMiner software.

For each model, the weights of all the metabolites selected to explain the class attribution were also evaluated by using a scree-plot-like graph, and the elbow of the graph was used as a threshold to identify the most relevant metabolites. These metabolites were combined in the Upset representation [61] to evaluate the selected metabolites from different models.

As part of the model training procedures, each model was subjected to cross validation. Furthermore, hyperparameter optimizations were also subjected to cross validation. To avoid overfitting of the trained models, the two cross-validations were nested in a single process to both train the models and tune the hyperparameters in an unbiased manner.

The 10 individual classification models were also “ensembled” according to a voting scheme that used both the cross-validation accuracy and the confidence (i.e., distance from classification margin) as a vote weight. Ensembling was executed in accordance with Troisi et al. [56,57,62,63,64,65]. In brief, for samples identified as “CRC”, the scores (obtained by multiplying the model cross-validation accuracy and classification confidence) were used as is, whereas the scores of “CTRL” samples were multiplied by −1. Lastly, the sum of the individual classification model scores was used to calculate a CRC-ensemble machine learning (EML) score for each subject. These scores were then compared to the optimized cut-off value to arrive at a final prediction of whether a given metabolome derived from a CTRL or CRC sample. The overall diagnostic performance of the proposed score was investigated using a confusion matrix to summarize the results obtained using samples in the test set.

The area under receiver operating characteristic (AUC-ROC) curve, as well as sensitivity, specificity, positive and negative predictive values, positive and negative likelihood ratios, and accuracy were calculated to assess the ability of the CRC-EML score to correctly predict CRC presence. A non-parametric approach (DeLong et al. [66]) was used to compare the AUC-ROC curves.

### 4.6. Pathway Analysis

The pathway analysis was performed combining the results from pathway enrichment analysis with pathway topology analysis according to Xia and Wishart [67]. The analysis was based on the KEGG metabolic pathways as the backend knowledgebase included in the web application based on the MetPa algorithm.

By means of the over-representation analysis, we first tested if compounds involved in a particular pathway were enriched compared to random hits. This evaluation was based on the hypergeometric test.

Moreover, to take into consideration the structural information of the pathways, a pathway topology analysis was performed using the betweenness centrality as a measure of node centrality to estimate node importance.

Because several pathways were tested at the same time, the statistical *p* values from enrichment analysis were adjusted for multiple testing with the false discovery rate (FDR) method. The Impact is the pathway impact value calculated from pathway topology analysis.

## 5. Conclusions

The present pilot study allowed the identification of a complex network of serum metabolites significantly associated with the presence of colorectal cancer. The metabolomic signature appears to be strongly correlated with the Warburg effect and glutamine addiction that are widely reported in several cancers. However, several aspects of this signature seem more specific to CRC; namely, oleamide and SCFA imbalances play a large role in determining the high specificity of this signature. Other studies are necessary to validate these preliminary results and to evaluate the specifics of the signature in the differentiation of CRC from other cancer types.

## Figures and Tables

**Table 1:** Enrolled subject characteristics (mean ± standard deviation or %). Abbreviations used are HS: Healthy subjects, BCRT: Benign colon or rectum tumors, CRC: Colorectal cancer affected patients, BMI: Body mass index, HDL: High-density lipoprotein, LDL: Low-density lipoprotein, GGT: Gamma-glutamyltransferase, AST: Aspartate aminotransferase, ALT: Alanine transaminase LDH: Lactate dehydrogenase.

	HS (*n* = 50)	BCRT (*n* = 50)	CRC (*n* = 100)
Age (years)	61.6 ± 7.0	62.8 ± 7.1	66.2 ± 11.3 *
Men (%)	56	59	64
Weight (kg)	76.4 ± 15.5	80.0 ± 16.9	72.8 ± 15.1 ^§^
Height (cm)	165.0 ± 9.5	167.5 ± 8.7	167.7 ± 9.4
BMI (kg/cm^2^)	27.9 ± 4.3	28.4 ± 4.8	25.7 ± 9.4 *^,§^
Blood Pressure (mm Hg)			
Systolic	135.2 ± 24.4	132.3 ± 17.7	139.9 ± 17.4
Diastolic	81.6 ± 11.4	81.9 ± 11.1	80.7 ± 8.0
Heart rate (bmp)	79.7 ± 7.7	79.8 ± 6.8	79.4 ± 7.5
Oxygen saturation (%)	99.0 ± 1.5	98.8 ± 1.6	99.7 ± 10.0
Azotemia (g/dL)	38.4 ± 10.4	40.8 ± 18.8	43.2 ± 13.5 *
Total Cholesterol (mg/dL)	191.9 ± 39.1	194.9 ± 42.2	189.2 ± 40.0
HDL (mg/dL)	57.2 ± 13.1	52.5 ± 14.7	62.9 ± 18.8 ^§^
LDL (mg/dL)	114.2 ± 30.1	113.7 ± 33.9	113.9 ± 33.9
Triglycerides (mg/dL)	115.4 ± 57.3	138.4 ± 95.3	116.6 ± 63.5
Creatinine (mg/dL)	0.8 ± 0.2	0.9 ± 0.4	0.9 ± 0.3 *
Alkaline phosphatase (UI/L)	53.7 ± 16.2	55.2 ± 13.3	81.4 ± 59.3 *^,§^
GGT (U/L)	26.4 ± 17.5	26.7 ± 22.4	49.2 ± 118.6
Glycaemia (mg/dL)	92.1 ± 25.3	99.1 ± 29.3	101.0 ± 26.7
White blood cells (n/µL)	6725.6 ± 1917.1	8427.6 ± 12315.9	6033.3 ± 1928.7 *
Red blood cells (n/µL)	4.97 * 10^6^ ± 4.92 * 10^6^	4.95 * 10^6^ ± 7.14 * 10^6^	4.66 * 10^6^ ± 6.78 * 10^6^ *
AST (mU/mL)	21.5 ± 7.7	23.8 ± 11.6	25.2 ± 11.9 *
ALT (mU/mL)	25.1 ± 12.2	28.1 ± 14.9	27.2 ± 16.9
LDH (U/L)	169.3 ± 27.1	177.6 ± 28.9	172.3 ± 34.9
Serum iron (µg/dL)	95.0 ± 33.6	95.5 ± 36.9	79.6 ± 42.8 *^,§^
Uric acid (mg/dL)	5.4 ± 1.4	5.8 ± 1.7	5.2 ± 1.4 ^§^
Other pathologies (n(%))	45 (90%)	40 (80%)	98 (98%)
Hypertension ^¶^	24 (53%)	25 (63%)	49 (50%)
Diabetes ^¶^	9 (20%)	8 (20%)	14 (14%)
Hypertriglyceridemia ^¶^	2 (4%)	1 (3%)	0 (0%)
Hypercholesterolemia ^¶^	6 (13%)	6 (15%)	4 (4%)
Heart disease ^¶^	6 (13%)	5 (13%)	7 (7%)
Cancer in other organ ^¶^	6 (13%)	3 (8%)	13 (13%)
Other ^¶^	11 (24%)	10 (25%)	24 (24%)
Pharmacological treatments (n(%))	39 (78%)	37 (74%)	82 (82%)

* Indicates statistical difference (*p* < 0.05) compared to HS; ^§^ indicates statistical differences (*p* < 0.05) compared to BCRT; ^¶^ indicates the percentage based on the cases with other pathologies.

**Table 2:** Performance metrics (value ± standard error) of the individual and the ensembled machine learning classification algorithms when applied to the test set. Abbreviations; NB: Naïve Bayes, GLM: Generalized linear model, LR: Logistic regression, FLM: Fast large margin, DL: Deep learning, DT: Decision tree, RF: Random forest, GBT: Gradient boosted tree, SVM: Support vector machine, PLS-DA: Partial least square discriminant analysis, EML: Ensemble machine learning, S: Sensitivity, Sp: Specificity; PLR: Positive likelihood ratio, NLR: Negative likelihood ratio, NPV: Negative predictive value, PPV: Positive predictive value, A: Accuracy, ND: Not determinable.

Model	S	Sp	PLR	NLR	NPV	PPV	A
NB	0.58 ± 0.10	1.00 ± 0.00	ND	0.42	0.67 ± 0.08	1.00 ± 0.00	0.77
GLM	0.96 ± 0.04	1.00 ± 0.00	ND	0.04	0.96 ± 0.04	1.00 ± 0.00	0.98
LR	0.88 ± 0.06	0.95 ± 0.05	18.58	0.12	0.87 ± 0.07	0.96 ± 0.04	0.91
FLM	1.00 ± 0.00	0.77 ± 0.09	4.40	0.00	1.00 ± 0.00	0.83 ± 0.07	0.89
DL	1.00 ± 0.00	1.00 ± 0.00	ND	0.00	1.00 ± 0.00	1.00 ± 0.00	1.00
DT	1.00 ± 0.00	0.82 ± 0.08	5.50	0.00	1.00 ± 0.00	0.86 ± 0.06	0.91
RF	0.69 ± 0.09	1.00 ± 0.00	ND	0.31	0.72 ± 0.08	1.00 ± 0.00	0.83
GBT	0.46 ± 0.10	1.00 ± 0.00	ND	0.54	0.61 ± 0.08	1.00 ± 0.00	0.71
SVM	0.81 ± 0.08	1.00 ± 0.00	ND	0.19	0.81 ± 0.08	1.00 ± 0.00	0.89
PLS-DA	0.92 ± 0.05	0.87 ± 0.06	7.10	0.10	0.90 ± 0.05	0.89 ± 0.05	0.90
EML	1.00 ± 0.00	1.00 ± 0.00	ND	0.00	1.00 ± 0.00	1.00 ± 0.00	1.00

## Data Availability

The data that support the findings of this study are available from the corresponding author, upon reasonable request. The data are not publicly available due to privacy.

## Supplementary Materials

The following are available online at https://www.mdpi.com/article/10.3390/metabo12020110/s1. Figure S1: Deconvoluted chromatograms of a control and a colorectal cancer serum sample, Figure S2: Box and Whisker plot of the raw and transformed data related to the relevant metabolites, Table S1: Metabolite subsets selected by genetic algorithm and used to train the final models, Table S2: Classification confidence for each model, and resulting final EML score, for each subject in the test set, Table S3: Metabolites selected as relevant according to VIP-score criterion (>2.0), volcano plot (2 < FC < 0.5 and *p*-value < 0.05), and UpSet plot, Table S4: Metabolite-set Enrichment Pathways analysis results derived from the metabolites selected by VIP-score and Volcano plot in CTRL vs. CRC comparison.
