# Supplementary material for: A Metabolomics-Based Screening Proposal for Colorectal Cancer
Source: Metabolites. 2022 Jan 25;12(2):110. doi: 10.3390/metabo12020110 (PMC8878838; doi:10.3390/metabo12020110)

## Slide 1
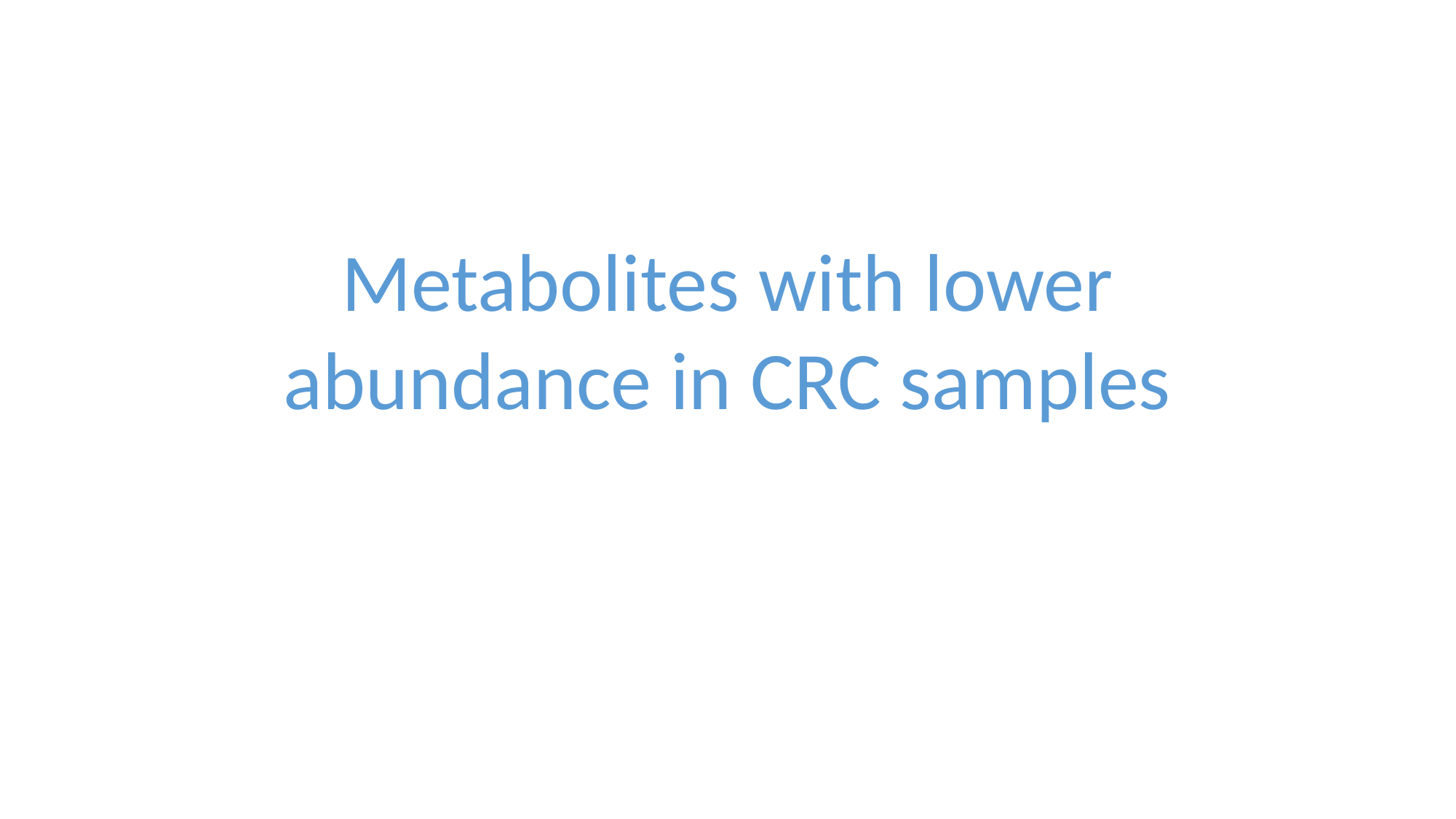

Metabolites with lower abundance in CRC samples

## Slide 2
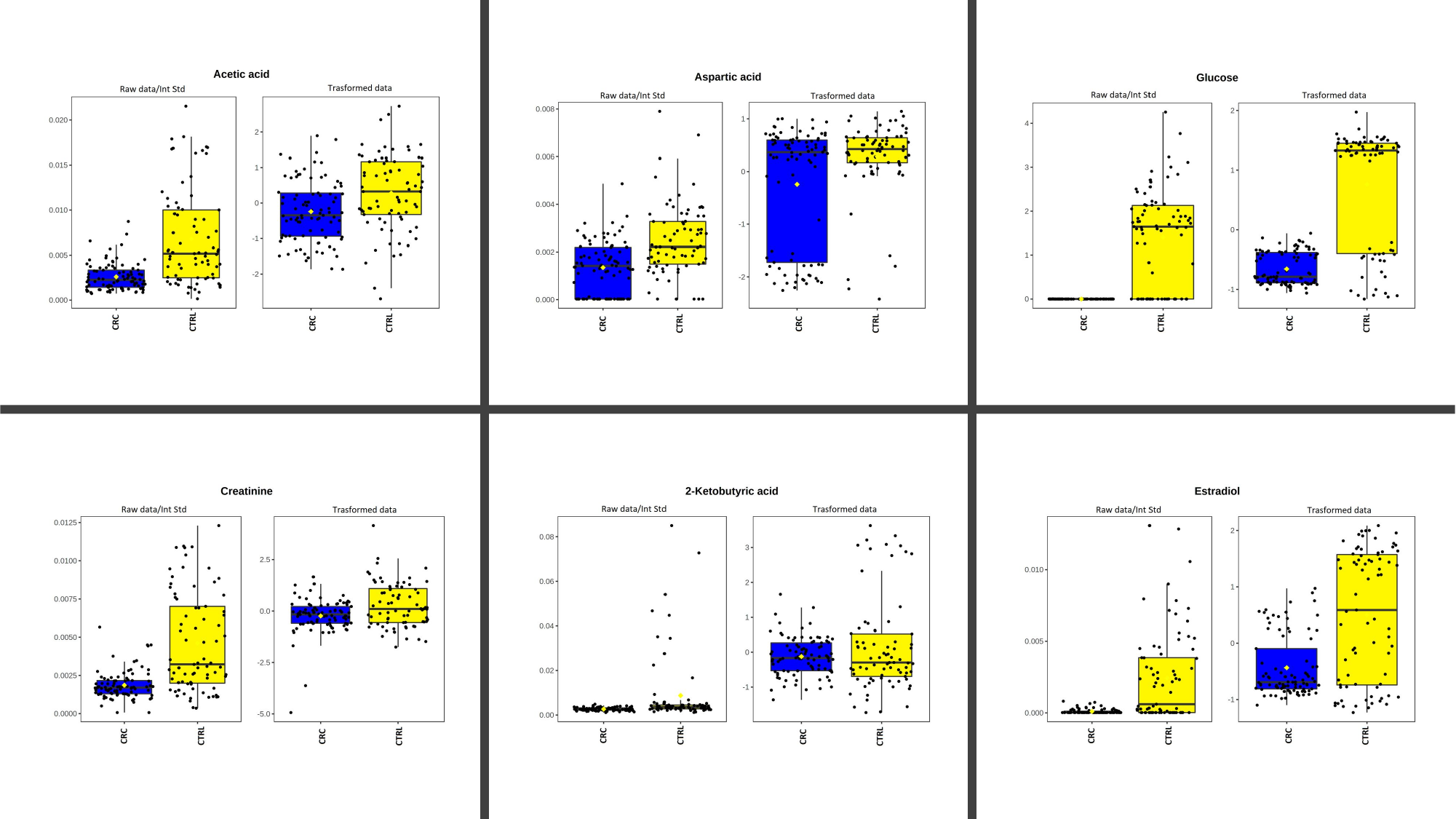

## Slide 3
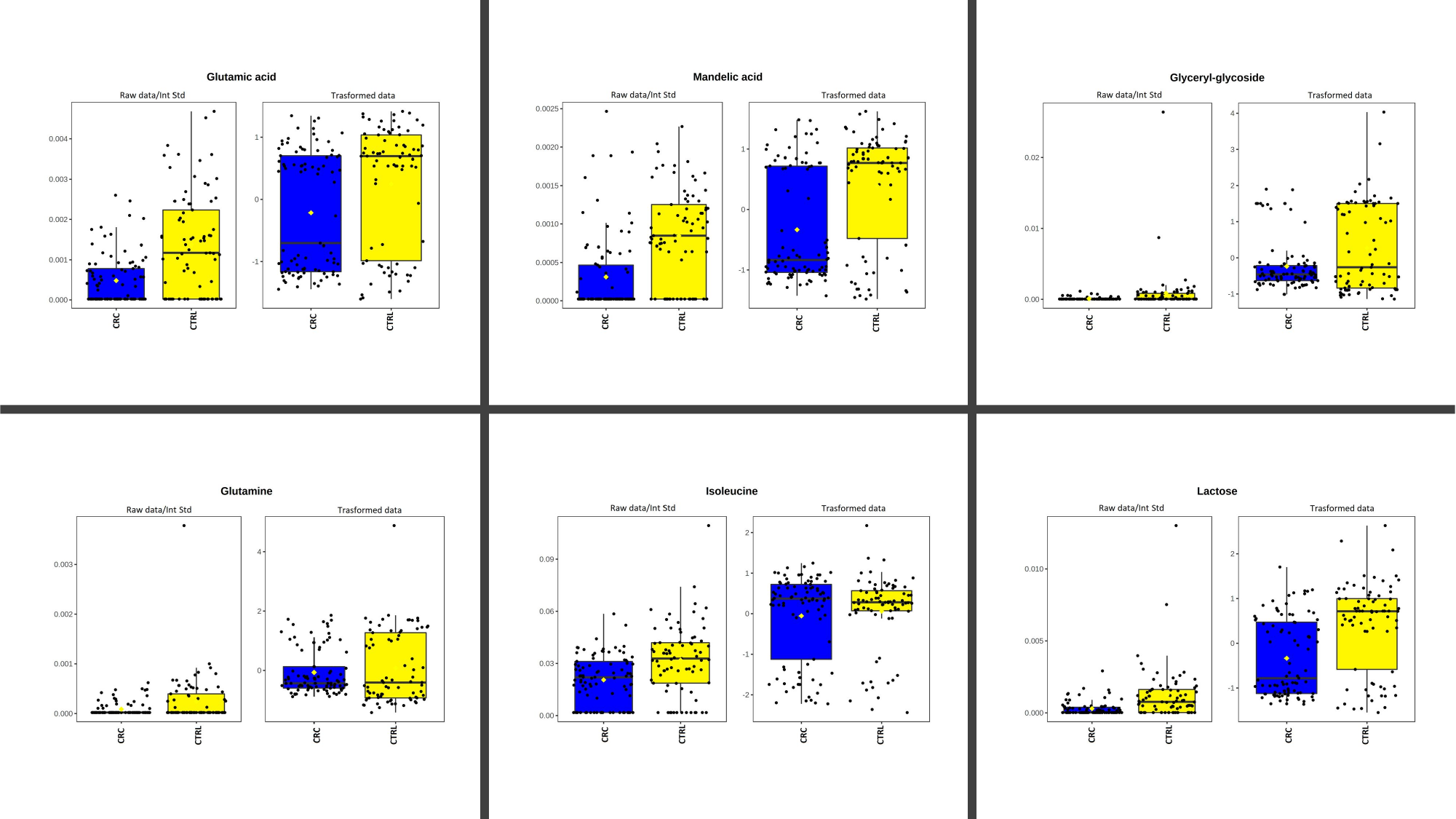

## Slide 4
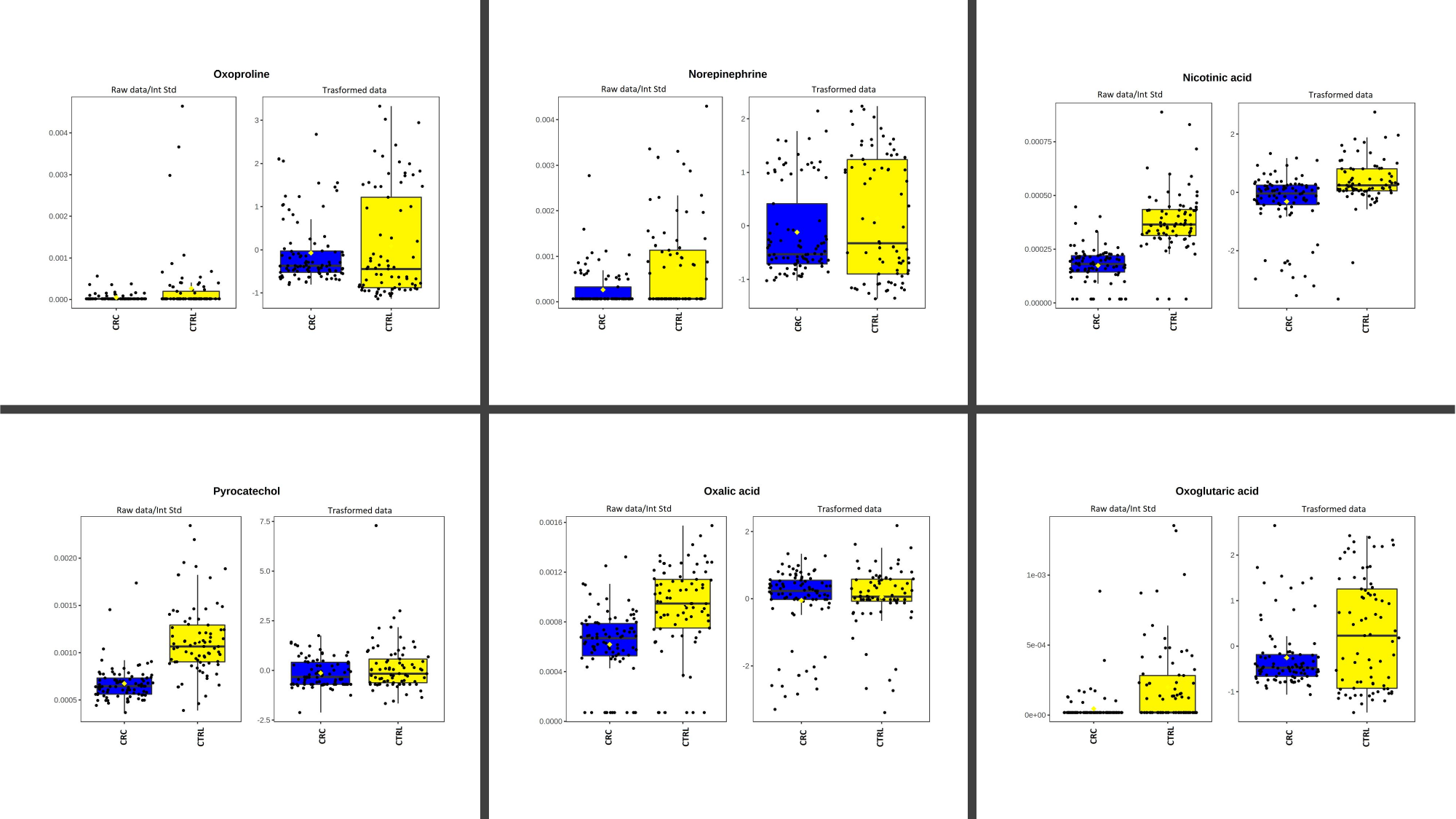

## Slide 5
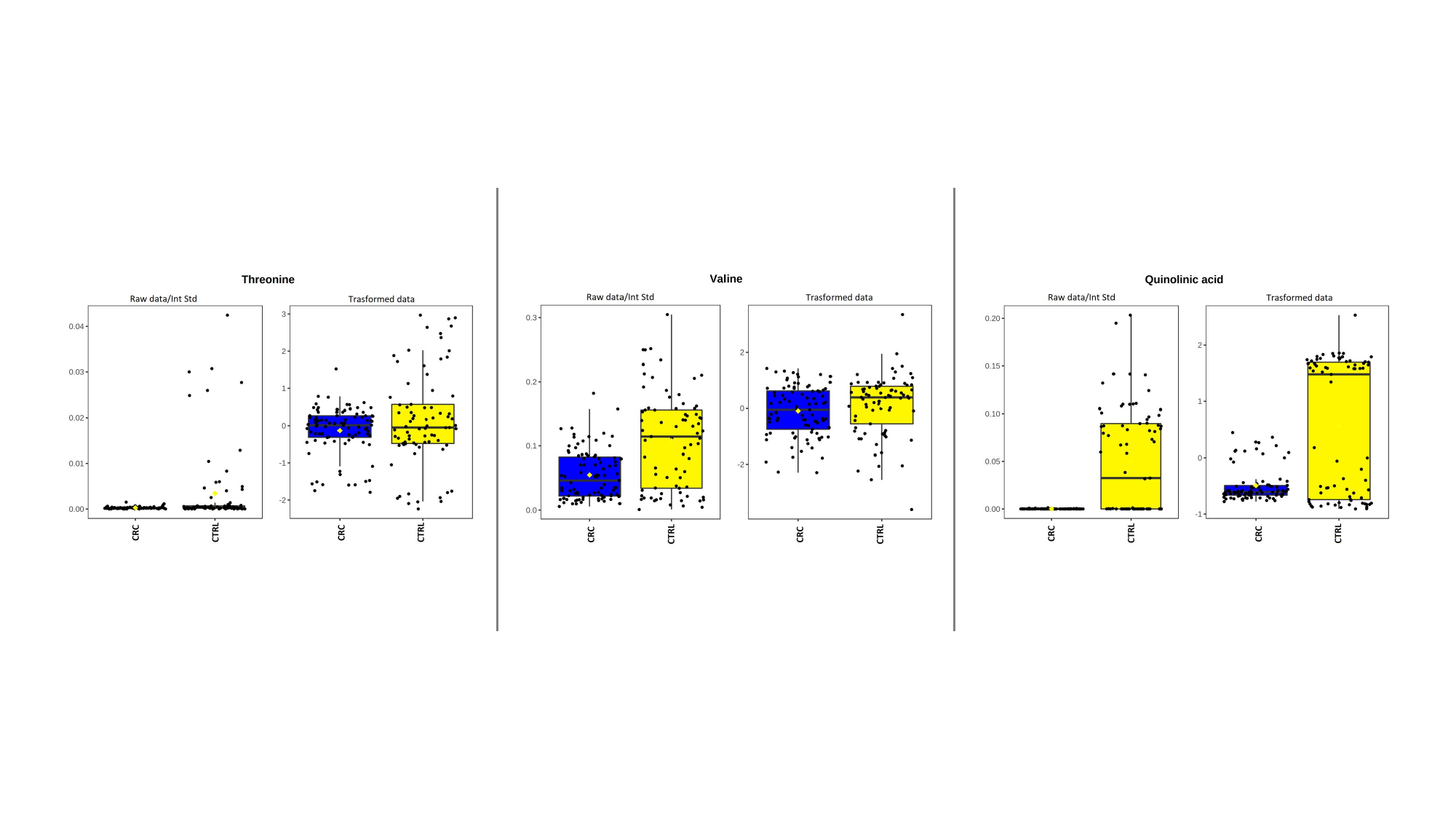

## Slide 6
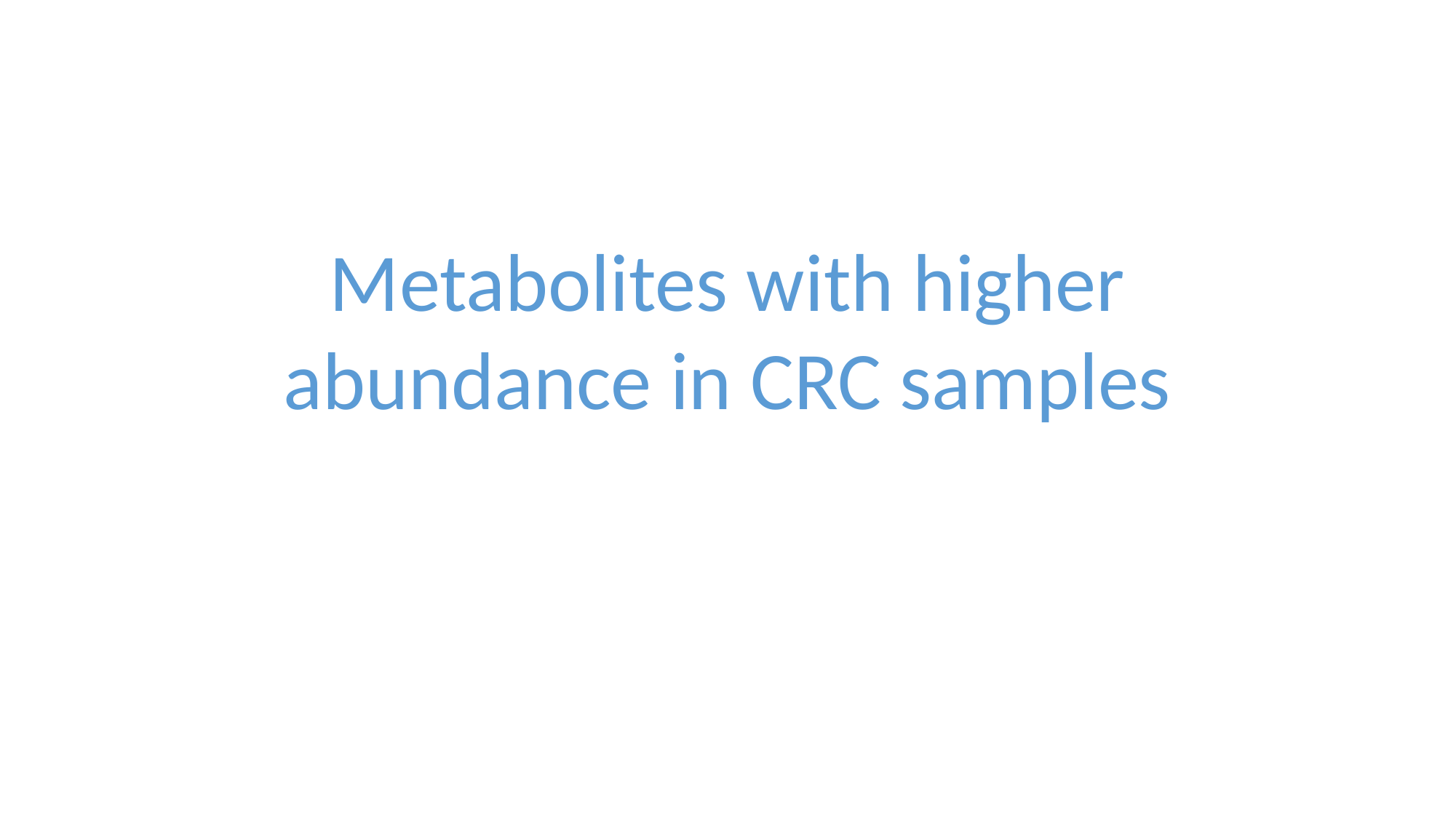

Metabolites with higher abundance in CRC samples

## Slide 7
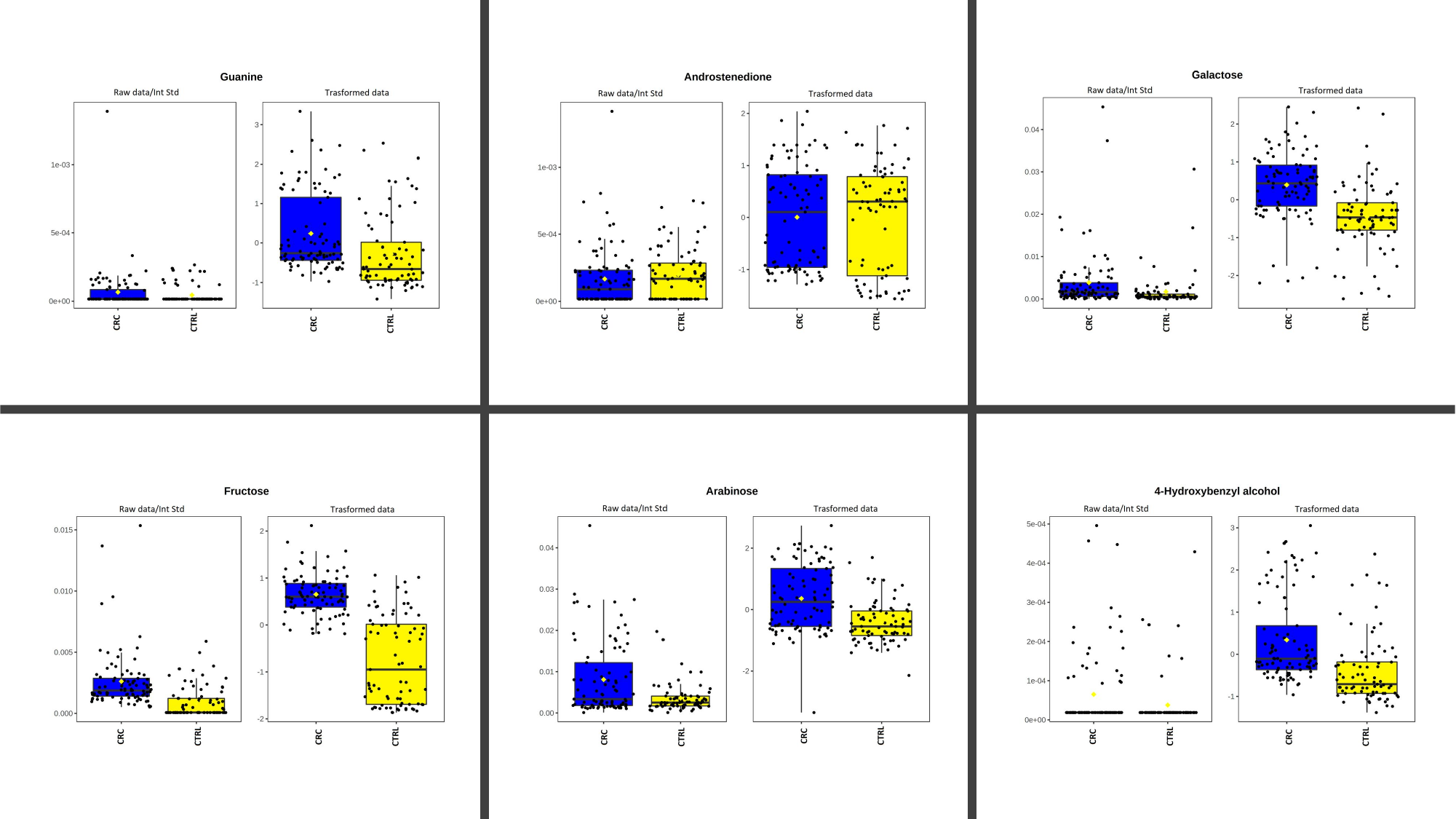

## Slide 8
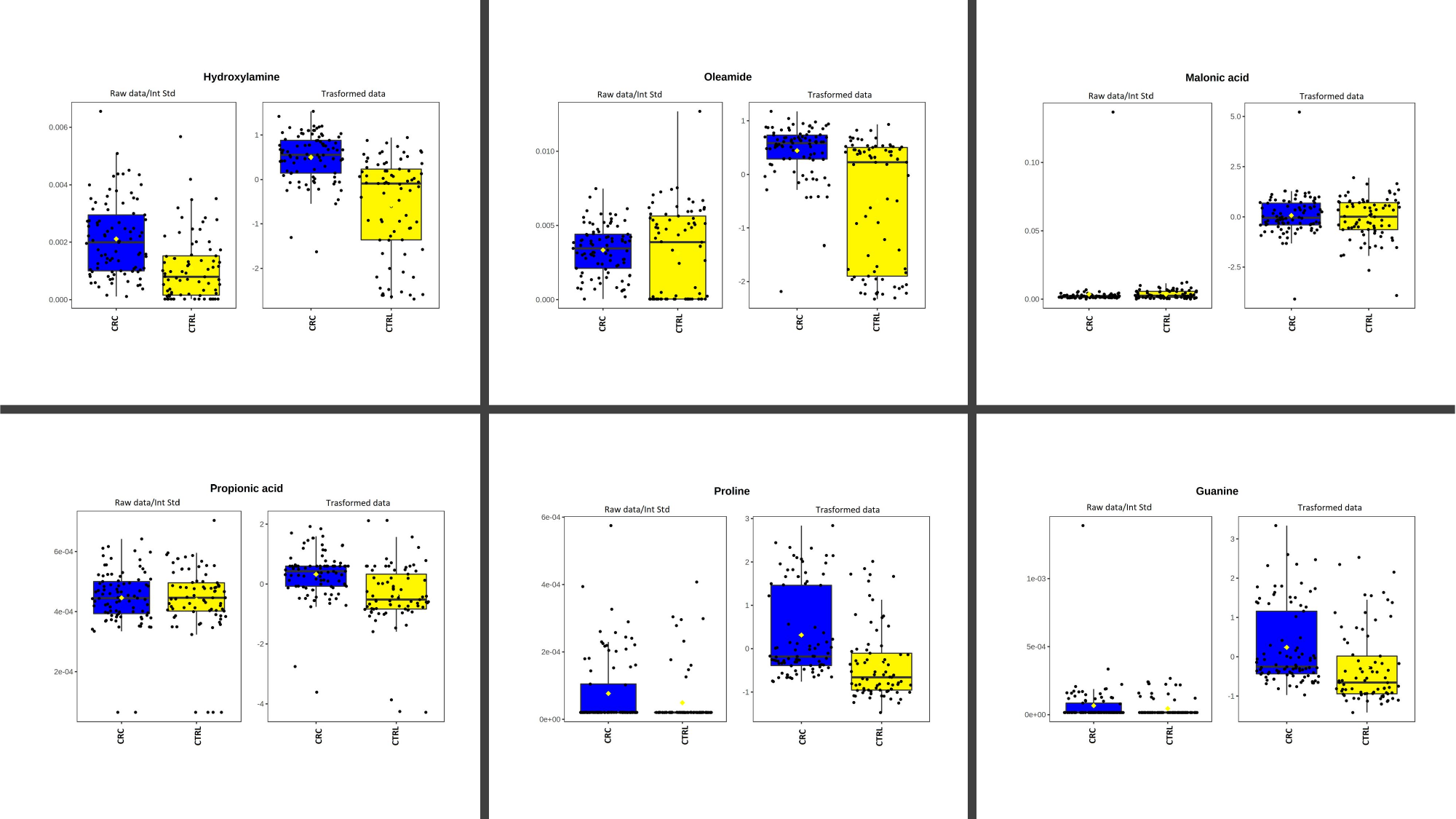

## Slide 9
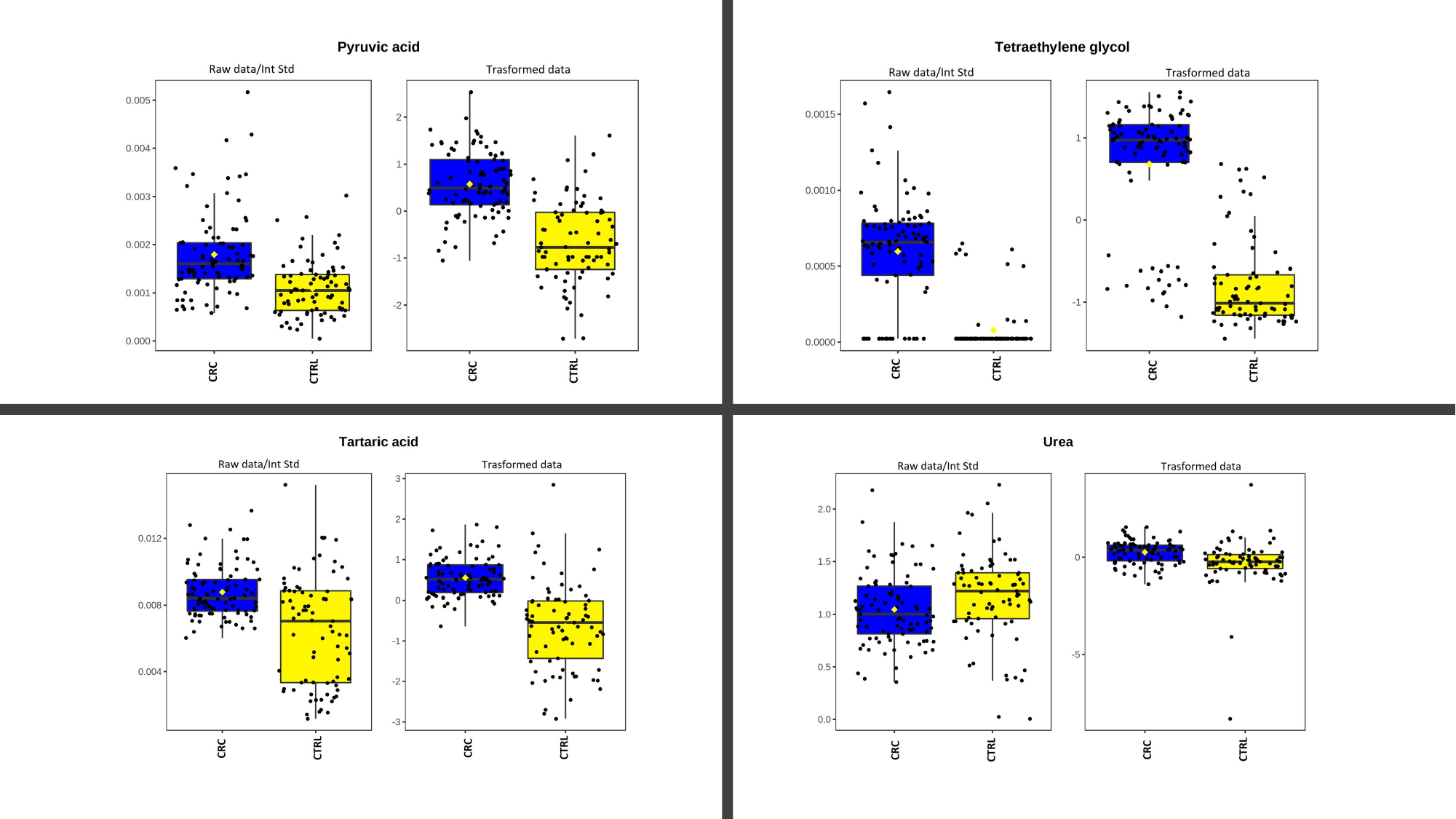

Supplement: Supplementary file 1 [file metabolites-12-00110-s001.zip › Figure S2.pptx]
