# Supplementary material for: A Metabolomics-Based Screening Proposal for Colorectal Cancer
Source: Metabolites. 2022 Jan 25;12(2):110. doi: 10.3390/metabo12020110 (PMC8878838; doi:10.3390/metabo12020110)
Supplement: Supplementary file 1 [file metabolites-12-00110-s001.zip › Table S1.pdf]

Table S1: Metabolites selected as relevant from the 10 trained classification models and from volcano plot evaluation. Human Metabolites Data Base (HMDB), Kyoto Encyclopedia of Genes and Genomes (KEGG), Chemical Entities of Biological Interest (ChEBI), and PubChem identification numbers were reported as well as the Simplified Molecular Input Line Entry System (SMILES) structures. (NA: Not Available)

Query	HMDB	PubChem	ChEBI	KEGG	SMILES
Glucose	HMDB0000516	64689	15903	C00221	OC[C@H]1O[C@@H](O)[C@H](O)[C@@H](O)[C@@H]1O
Nicotinic acid	HMDB0001488	938	15940	C00253	OC(=O)C1=CN=CC=C1
Tetra ethylene glycol	NA	8200	44920	NA	C(COCCOCCOCCO)O
Quinolinic acid	HMDB0000232	1066	16675	C03722	OC(=O)C1=CC=CN=C1C(O)=O
Myristic acid	HMDB0000806	11005	28875	C06424	CCCCCCCCCCCCCC(=O)O
Estradiol	HMDB0000151	5757	16469	C00951	C[C@]12CC[C@H]3[C@H]([C@@H]1CC[C@@H]2O)CCC4=C3C=CC(=C4)O
Lactose	HMDB0041627	6134	36218	C01970	[H][C@@]1(O)O[C@]([H])(CO)[C@@]([H])(O[C@]2([H])O[C@]([H])(CO)[C@]([H])(O)[C@]([H])(O)[C@@]2([H])O)[C@]([H])(O)[C@@]1([H])O
Androstenedione	HMDB0000053	6128	16422	C00280	C[C@]12CCC(=O)C=C1CC[C@@H]3[C@@H]2CC[C@]4([C@H]3CCC4=O)C
Acetic acid	HMDB0000042	176	15366	C00033	CC(=O)O
Hydroxylamine	HMDB0003338	787	15429	C00192	NO
Urea	HMDB0000294	1176	16199	C00086	NC(N)=O
Glutamine	HMDB0000641	5961	18050	C00064	N[C@@H](CCC(N)=O)C(O)=O
Oxoglutaric acid	HMDB0000208	51	30915	C00026	OC(=O)CCC(=O)C(O)=O
Oxalic acid	HMDB0002329	971	16995	C00209	OC(=O)C(O)=O
Tartaric acid	HMDB0000956	444305	15671	C00898	O[C@H]([C@@H](O)C(O)=O)C(O)=O
Fructose	HMDB0000660	439709	28645	C02336	OC[C@H]1O[C@](O)(CO)[C@@H](O)[C@@H]1O
Norepinephrine	HMDB0000216	439260	18357	C00547	NC[C@H](O)C1=CC(O)=C(O)C=C1
Guanine	HMDB0000132	764	16235	C00242	NC1=NC(=O)C2=C(N1)N=CN2
Propionic acid	HMDB0000237	1032	30768	C00163	CCC(=O)O
Valine	HMDB0000883	6287	16414	C00183	CC(C)[C@H](N)C(O)=O
Pyruvic acid	HMDB0000243	1060	32816	C00022	CC(=O)C(=O)O
Isoleucine	HMDB0000557	99288	43433	NA	CC[C@@H](C)[C@H](N)C(O)=O
Pyrocatechol	HMDB0000957	289	18135	C15571	OC1=CC=CC=C1O
Oxoproline	HMDB0000267	7405	18183	C01879	OC(=O)[C@@H]1CCC(=O)N1
Threonine	HMDB0000167	6288	16857	C00188	C[C@@H](O)[C@H](N)C(O)=O
Aspartic acid	HMDB0000191	5960	17053	C00049	N[C@@H](CC(O)=O)C(O)=O

Creatinine	HMDB0000562	588	16737	C00791	CN1CC(=O)NC1=N
Glutamic acid	HMDB0000148	33032	16015	C00025	N[C@@H](CCC(O)=O)C(O)=O
Mandelic acid	HMDB0000703	439616	32800	C01984	O[C@H](C(O)=O)C1=CC=CC=C1
Arabinose	HMDB0000646	439195	17535	C00259	O[C@H]1COC(O)[C@H](O)[C@H]1O
Galactose	HMDB0000143	439357	28061	C00984	OC[C@H]1O[C@H](O)[C@H](O)[C@@H](O)[C@H]1O
Proline	HMDB0000162	145742	17203	C00148	OC(=O)[C@@H]1CCCN1
Oleamide	HMDB0002117	5283387	116314	C19670	CCCCCCCC/C=C\CCCCCCCC(=O)N
4-Hydroxybenzyl alcohol	HMDB0011724	125	67410	C17467	OCC1=CC=C(O)C=C1
Palmitic acid	HMDB0000220	985	15756	C00249	CCCCCCCCCCCCCCCC(=O)O
Glyceryl-glycoside	NA	NA	24406	NA	NA
Malonic acid	HMDB0000691	867	30794	C04025	O=C(O)CC(=O)O
2-Ketobutyric acid	HMDB0000005	58	30831	C00109	CCC(=O)C(O)=O
