# Supplementary material for: A Metabolomics-Based Screening Proposal for Colorectal Cancer
Source: Metabolites. 2022 Jan 25;12(2):110. doi: 10.3390/metabo12020110 (PMC8878838; doi:10.3390/metabo12020110)
Supplement: Supplementary file 1 [file metabolites-12-00110-s001.zip › Table S2.pdf]

Table S2: Metabolite-set Enrichment Pathways analysis results derived from the metabolites selected as relevant in the classification of CTRL vs. CRC serum samples. Total column represents the total number of compounds in the pathway; the Hits column is the number of metabolites from a given pathway that were selected as relevant; the Raw p is the original p value evaluated from the enrichment analysis; the Holm adjust column is the raw p-value adjusted by the Holm-Bonferroni method; the FDR is the p-value adjusted using False Discovery Rate; the Impact is the pathway impact value calculated from pathway topology analysis.

	**Total**	**Expected**	**Hits**	**Raw p**	**-Log$_{10}$(p)**	**Holm adjust**	**FDR**	**Impact**
Arginine biosynthesis	14	0.32516	5	8.75E-06	5.0578	0.000735	0.000676	0.11675
Valine, leucine and isoleucine biosynthesis	8	0.18581	4	1.61E-05	4.7932	0.001336	0.000676	0
Aminoacyl-tRNA biosynthesis	48	1.1148	7	7.37E-05	4.1324	0.006045	0.002064	0
D-Glutamine and D-glutamate metabolism	6	0.13935	3	0.00022	3.6583	0.017791	0.004613	0.5
Alanine, aspartate and glutamate metabolism	28	0.65032	5	0.000339	3.4694	0.027145	0.005701	0.58254
Nicotinate and nicotinamide metabolism	15	0.34839	3	0.004324	2.3641	0.34163	0.060542	0
Glyoxylate and dicarboxylate metabolism	32	0.74323	4	0.005547	2.256	0.43265	0.066562	0
Nitrogen metabolism	6	0.13935	2	0.007422	2.1295	0.57148	0.077929	0
Galactose metabolism	27	0.6271	3	0.022957	1.6391	1	0.21427	0.19819
Glycine, serine and threonine metabolism	33	0.76645	3	0.038938	1.4096	1	0.32149	0
Butanoate metabolism	15	0.34839	2	0.045562	1.3414	1	0.32149	0
Neomycin, kanamycin and gentamicin biosynthesis	2	0.046452	1	0.045927	1.3379	1	0.32149	0
Histidine metabolism	16	0.37161	2	0.051323	1.2897	1	0.33163	0
Arginine and proline metabolism	38	0.88258	3	0.055637	1.2546	1	0.33382	0.1638
Pantothenate and CoA biosynthesis	19	0.44129	2	0.070037	1.1547	1	0.39221	0
Citrate cycle (TCA cycle)	20	0.46452	2	0.076707	1.1152	1	0.40271	0.1049
Pyruvate metabolism	22	0.51097	2	0.090621	1.0428	1	0.44778	0.26749
Propanoate metabolism	23	0.53419	2	0.097841	1.0095	1	0.45659	0.04061
Glycolysis / Gluconeogenesis	26	0.60387	2	0.12042	0.91931	1	0.53238	0.1295
Glutathione metabolism	28	0.65032	2	0.13613	0.86604	1	0.57175	0.02675
Cysteine and methionine metabolism	33	0.76645	2	0.17718	0.75157	1	0.70874	0.05983
Purine metabolism	65	1.5097	3	0.18925	0.72297	1	0.72259	0.01281
Amino sugar and nucleotide sugar metabolism	37	0.85935	2	0.21131	0.67508	1	0.77173	0
Valine, leucine and isoleucine degradation	40	0.92903	2	0.23735	0.62461	1	0.83072	0

Tyrosine metabolism	42	0.97548	2	0.25483	0.59375	1	0.85623	0.08872
Fatty acid biosynthesis	47	1.0916	2	0.29865	0.52484	1	0.96486	0.01473
Pentose and glucuronate interconversions	18	0.41806	1	0.34647	0.46034	1	1	0
Starch and sucrose metabolism	18	0.41806	1	0.34647	0.46034	1	1	0.4207
beta-Alanine metabolism	21	0.48774	1	0.39149	0.40728	1	1	0
Porphyrin and chlorophyll metabolism	30	0.69677	1	0.50922	0.2931	1	1	0
Biosynthesis of unsaturated fatty acids	36	0.83613	1	0.57506	0.24029	1	1	0
Steroid hormone biosynthesis	85	1.9742	2	0.59754	0.22363	1	1	0.14685
Fatty acid elongation	39	0.90581	1	0.60468	0.21848	1	1	0
Fatty acid degradation	39	0.90581	1	0.60468	0.21848	1	1	0
Pyrimidine metabolism	39	0.90581	1	0.60468	0.21848	1	1	0
