# Supplementary figures and images for: A Metabolomics-Based Screening Proposal for Colorectal Cancer
Source: Metabolites. 2022 Jan 25;12(2):110. doi: 10.3390/metabo12020110 (PMC8878838; doi:10.3390/metabo12020110)

## Slide 1
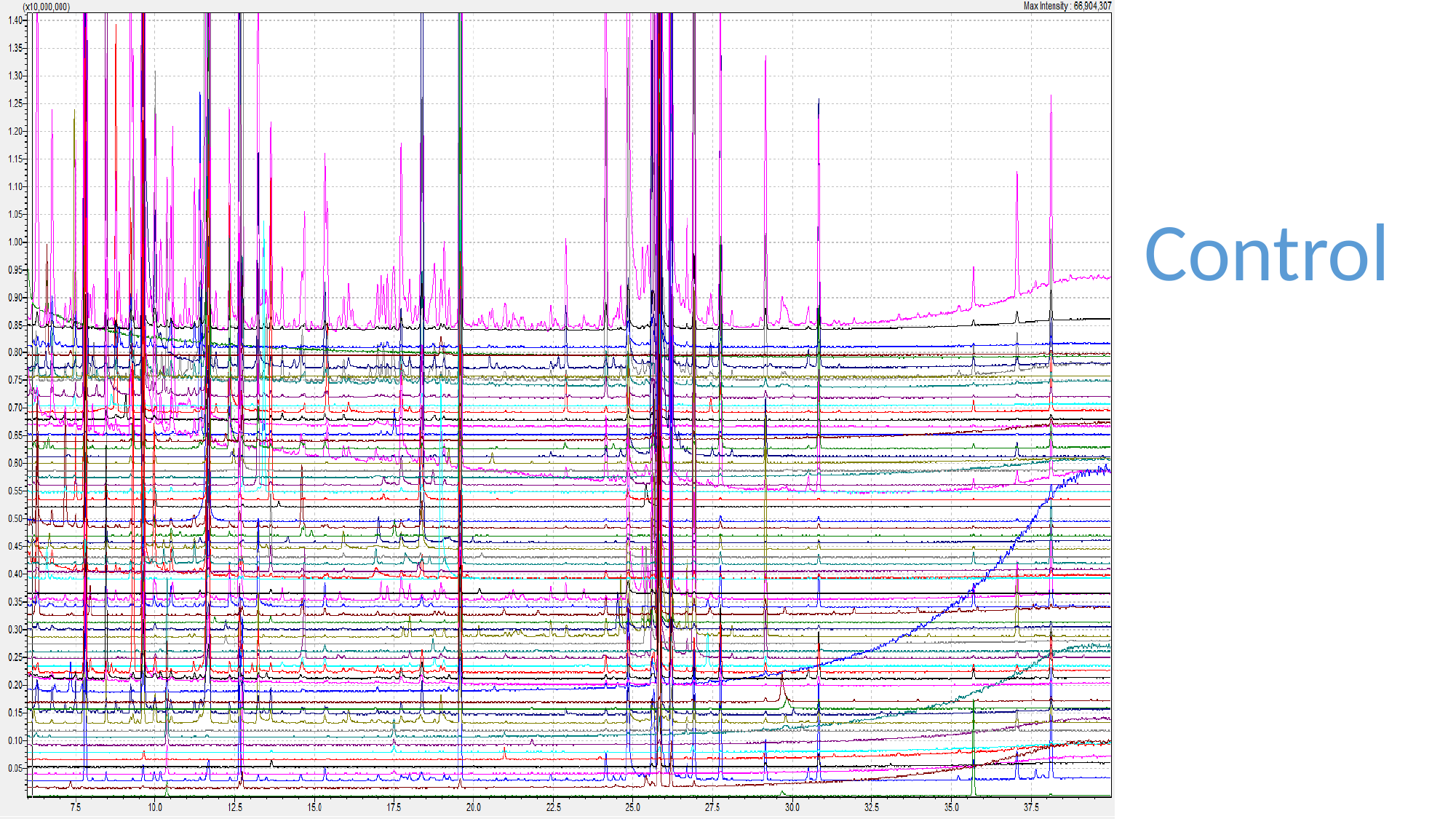

Control

## Slide 2
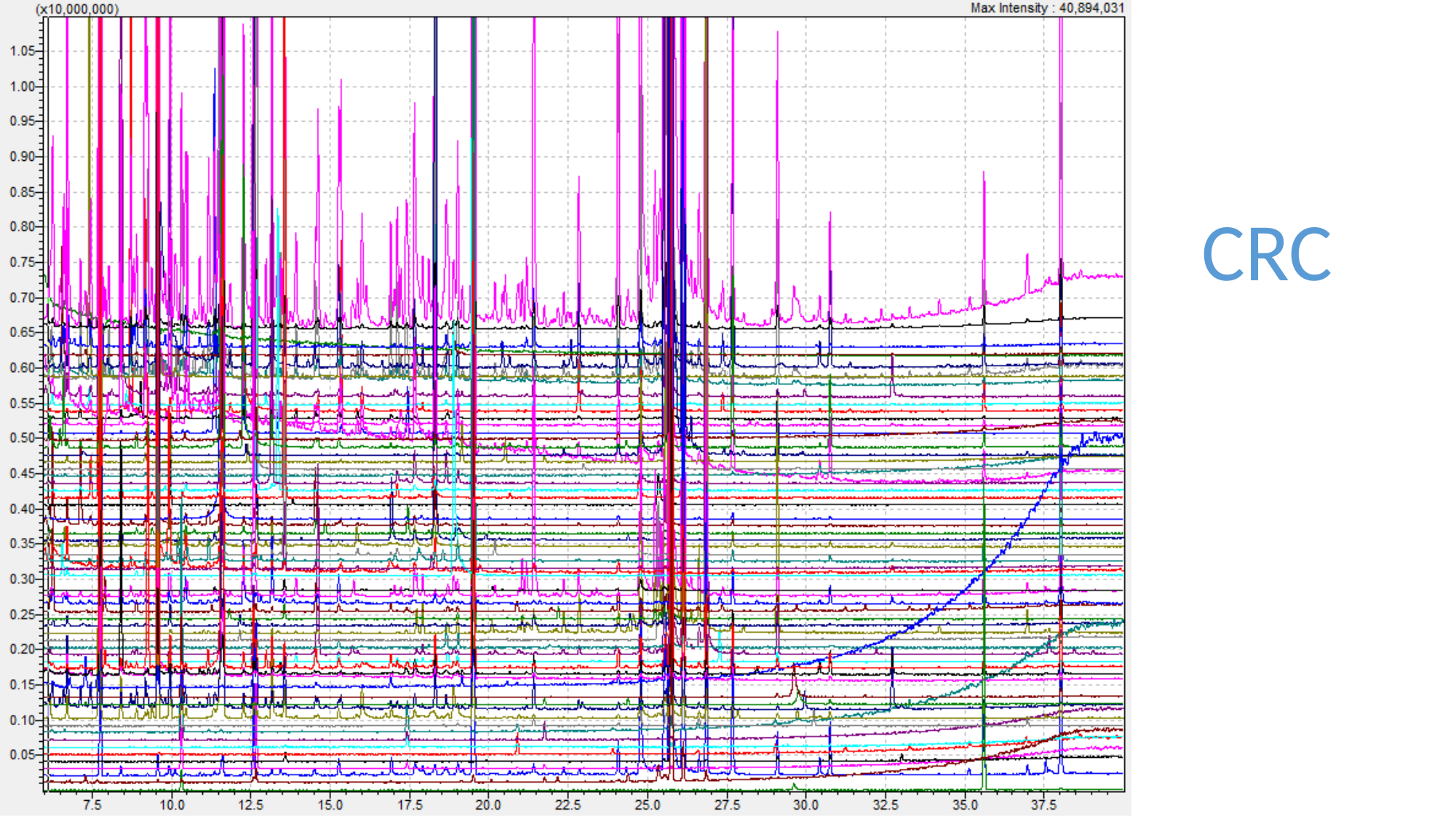

CRC

Supplement: Supplementary file 1 [file metabolites-12-00110-s001.zip › Figure S1.pptx]
